# Obesity and diabetes genes are associated with being born small for gestational age: Results from the Auckland Birthweight Collaborative study

**DOI:** 10.1186/1471-2350-11-125

**Published:** 2010-08-16

**Authors:** Angharad R Morgan, John MD Thompson, Rinki Murphy, Peter N Black, Wen-Jiun Lam, Lynnette R Ferguson, Ed A Mitchell

**Affiliations:** 1 Discipline of Nutrition, Faculty of Medical and Health Sciences, The University of Auckland, Auckland, New Zealand; 2 Nutrigenomics New Zealand, New Zealand; 3 Department of Paediatrics, Faculty of Medical and Health Sciences, The University of Auckland, Auckland, New Zealand; 4 Department of Medicine, Faculty of Medical and Health Sciences, The University of Auckland, Auckland, New Zealand; 5 Department of Pharmacology, Faculty of Medical and Health Sciences, The University of Auckland, Auckland, New Zealand

## Abstract

**Background:**

Individuals born small for gestational age (SGA) are at increased risk of rapid postnatal weight gain, later obesity and diseases in adulthood such as type 2 diabetes, hypertension and cardiovascular diseases. Environmental risk factors for SGA are well established and include smoking, low pregnancy weight, maternal short stature, maternal diet, ethnic origin of mother and hypertension. However, in a large proportion of SGA, no underlying cause is evident, and these individuals may have a larger genetic contribution.

**Methods:**

In this study we tested the association between SGA and polymorphisms in genes that have previously been associated with obesity and/or diabetes. We undertook analysis of 54 single nucleotide polymorphisms (SNPs) in 546 samples from the Auckland Birthweight Collaborative (ABC) study. 227 children were born small for gestational age (SGA) and 319 were appropriate for gestational age (AGA).

**Results and Conclusion:**

The results demonstrated that genetic variation in *KCNJ11, BDNF, PFKP, PTER *and *SEC16B *were associated with SGA and support the concept that genetic factors associated with obesity and/or type 2 diabetes are more prevalent in those born SGA compared to those born AGA. We have previously determined that environmental factors are associated with differences in birthweight in the ABC study and now we have demonstrated a significant genetic contribution, suggesting that the interaction between genetics and the environment are important.

## Background

Small for gestational age (SGA) babies (typically defined as birthweight below the 10^th ^centile according to gestational age [1,2]) are not only at increased risk of neonatal morbidity and mortality, but are also at increased risk of rapid postnatal weight gain, later obesity and diseases in adulthood such as type 2 diabetes, hypertension and ischemic heart disease [3-7] which are major causes of adult morbidity and mortality worldwide. Although the cause of this association is unknown, several hypotheses have been proposed. The fetal insulin hypothesis [8] proposes that common genes inherited by the fetus affect both birth size and predisposition to adult diseases. In contrast, the Barker hypothesis [4,5,9-11] suggests the association to be the result of fetal programming - permanent changes in physiology and metabolism in response to adverse maternal uterine environment in pregnancy that result in increased metabolic disease risk in adulthood. The increased risk of adult metabolic diseases in those who are born small at birth is further amplified by an accelerated pattern of growth during childhood. The thrifty phenotype hypothesis explains this phenomenon by suggesting that the fetal nutrient conserving adaptations in response to intrauterine under nutrition is overwhelmed by nutrient abundance post-natally and manifests in adult metabolic diseases [9]. Singhal and Lucas [12] propose that it is not low birth weight per se, but this rapid postnatal growth that is responsible for the increased risk for disease in later life.

Environmental risk factors for SGA are well established and include smoking, low pregnancy weight, maternal short stature, maternal diet, ethnic origin of mother and hypertension [13]. These risk factors have been studied and confirmed in the Auckland Birthweight Collaborative (ABC) study [14,15]. However, in a large proportion of SGA, no underlying cause is evident, and these individuals may have a larger genetic contribution. The existence of such genetic factors is supported by the observation that SGA births tend to cluster in families and to recur in successive generations [16,17]. However, genetic association publications for SGA to date have been rare and inconsistent. Infante-Rivard *et al*., 2007 [18] provides a summary overview of genetic association studies for SGA. In brief, only the thrombophilia pathway including genetic variants in *MTHFR, FV*, and *FII *[19,20], and xenobiotic-metabolizing pathways with variants in *CYP1A1, GSTT *and *GSTM *[21,22] have been studied with any frequency. Other pathways that have been studied with less frequency include the vascular dysfunction or atherosclerosis pathway with variants in *APOE, PON *and *ACE *[23-25] and the insulin resistance pathway with variants in *IGF-1 *[26]. However, none of these pathways have shown robust associations with susceptibility to adult diseases with which SGA has been linked.

As children born SGA often become obese in later life and/or develop type 2 diabetes we tested the association between SGA and polymorphisms in genes that have previously been associated with obesity and/or type 2 diabetes under the hypothesis that a common genetic denominator might predispose to both SGA and obesity and/or type 2 diabetes. The hypothesis was tested in subjects from the Auckland Birthweight Collaborative (ABC) study. Since we began this study there have been several publications examining the relationship between type 2 diabetes susceptibility genes and birthweight (these studies are described in the discussion), however we have included both obesity and type 2 diabetes susceptibility genes and we have focused on SGA status.

## Methods

### Samples

The ABC study has been fully described previously [14].

In summary, between 16 October 1995 and 12 August 1996, babies born and resident in the Waitemata Health or Auckland Healthcare regions were eligible for inclusion and from 12 August 1996 to 30 November 1997, babies born and resident in the Auckland Healthcare region were eligible for inclusion. Preterm infants (< 37 completed weeks of gestation), multiple births and those with congenital abnormalities were excluded. The sample was selected disproportionately to include all infants born at term and SGA (≤10th percentile for sex and gestation for New Zealand) [27] and a random sample of appropriate for gestational age (AGA) infants (> 10th percentile for sex and gestation). Gestational age was estimated using the date of the last menstrual period where it was available and was within 2 weeks of the best clinical estimate of gestational age at birth; otherwise the best clinical estimate was used.

Data have been collected at birth, 1, 3.5, 7 and most recently 11 years of age. The original sample at birth resulted in a sample of 1714 subjects, of which 871 mothers were identified in the obstetric data to be of European ethnicity. At the age of 1 and 3.5 follow up of non-European ethnicities was poor resulting in a lack of ability to generalise the results from these children to their particular populations. As a result follow-up from the age of 7 has only been carried out on those children whose mothers were identified as European ethnicity at birth. At 11 years 546 participants consented to collection of peripheral blood (n = 397) or a buccal swab (n = 149) for DNA extraction and genotyping. 227 samples were from children born small for gestational age (SGA) and 319 were from children born appropriate for gestational age (AGA).

DNA was extracted from the blood/buccal samples using Qiagen's DNA extraction kit and following the manufacturer's instructions.

The study received ethical approval from the Northern Regional Ethics Committee. Signed consent for the study and extraction of DNA was given by the parents of the children and assent also given by the child.

### SNP selection

The SNPs were selected from a systematic literature search to identify genetic variants demonstrating association with obesity and/or type 2 diabetes. We included 18 diabetes SNPs and 46 obesity SNPs identified from published candidate gene or genome-wide association studies, totalling 64 SNPs which were located in 42 genes. See table 1 for the list of genes/SNPs selected for investigation.

**Table 1:** Type 2 diabetes and obesity SNPs/genes we investigated for association with SGA in the Auckland Birthweight Collaborative study samples

Gene region	Chr	Association	Method of identification	References	SNP	Description
NOTCH2	1	Type 2 Diabetes	GWAS	[45]	rs10923931	Intronic
THADA	2	Type 2 Diabetes	GWAS	[45]	rs7578597	Missense: T1187A
ADAMTS9	3	Type 2 Diabetes	GWAS	[45]	rs4607103	38 kb upstream
IGF2BP2	3	Type 2 Diabetes	GWAS	[46-48]	rs4402960	Intronic
PPARG	3	Type 2 Diabetes	Candidate-gene studies	[49,50]	rs1801282	Missense: P12A
WFS1	4	Type 2 Diabetes	Candidate-gene studies	[51-53]	rs10010131	Intron-exon junction
CDKAL1	6	Type 2 Diabetes	GWAS	[46-48,54]	rs7754840	Intronic
JAZF1	7	Type 2 Diabetes	GWAS	[45]	rs864745	Intronic
SLC30A8	8	Type 2 Diabetes	GWAS	[47,48]	rs13266634	Missense: R325W
CDKN2A/B	9	Type 2 Diabetes	GWAS	[46-48]	rs10811661	125 kb upstream
CDC123-CAMK1D	10	Type 2 Diabetes	GWAS	[45]	rs12779790	Intergenic region
TCF7L2	10	Type 2 Diabetes	Fine-mapping of linkage peak	[38,55,56]	rs7903146	Intronic
HHEX	10	Type 2 Diabetes	GWAS	[46-48]	rs1111875	7.7 kb downstream
KCNJ11	11	Type 2 Diabetes	Candidate-gene studies	[52,57-59]	rs5219	Missense: E23K
MTNR1B	11	Type 2 Diabetes	GWAS	[60-62]	rs10830963	Intronic
					rs1387153	29 kb upstream
TSPAN8-LGR5	12	Type 2 Diabetes	GWAS	[45]	rs7961581	Intronic
HNF1B	17	Type 2 Diabetes	Candidate-gene studies	[63,64]	rs757210	Intronic
NEGR1	1	Obesity	GWAS	[65,66]	rs2568958	16.7 kb downstream
					rs2815752	64 kb downstream
SEC16B	1	Obesity	GWAS	[65]	rs10913469	Intronic
ADIPOR1	1	Obesity	GWAS	[67]	rs3820152	Intronic
TMEM18	2	Obesity	GWAS	[65,66]	rs7561317	23 kb upstream
					rs6548238	33 kb upstream
INSIG2	2	Obesity	GWAS	[68,69]	rs7566605	10 kb upstream
					rs2012693	25.8 kb upstream
ETV5 - DGKG	3	Obesity	GWAS	[65]	rs7647305	Intergenic
GNPDA2	4	Obesity	GWAS	[66]	rs10938397	453.9 kb downstream
NCR3 - AIF1	6	Obesity	GWAS	[65]	rs2844479	Intergenic
NAMPT	7	Obesity	Candidate gene study	[70]	rs10487818	Intronic
MTMR9	8	Obesity	GWAS	[71]	rs2293855	intronic
LPL	8	Obesity	GWAS	[67]	rs3200218	3'UTR
PTER	10	Obesity	GWAS	[72]	rs10508503	179 kb upstream
PFKP	10	Obesity	GWAS	[67,69]	rs6602024	Intronic
					rs2388395	317 kb upstream
					rs2388399	317 kb upstream
					rs2388397	317 kb upstream
MTCH2	11	Obesity	GWAS	[66]	rs10838738	Intronic
BDNF	11	Obesity	GWAS	[65]	rs6265	Missense V66M
					rs925946	9.2 kb upstream
ADIPOR2	12			[67]	rs2286385	Intronic
BCDIN3 D - FAIM2	12	Obesity	GWAS	[65]	rs7138803	Intergenic
MAF	16	Obesity	GWAS	[72]	rs1424233	48 kb downstream
SH2B1	16	Obesity	GWAS	[65,66]	rs7498665	Missense A434T
FTO	16	Obesity and Type 2 diabetes	GWAS	[65,67,73,74]	rs9939609	Intronic
					rs6499640	Intronic
					rs8050136	Intronic
NCP1	16	Obesity	GWAS	[72]	rs1805081	Missense H 215R
MC4R	18	Obesity	GWAS	[65,75,76]	rs17782313	187.5 kb upstream
					rs17700633	109.1 kb upstream
					rs12970134	153.8 kb upstream
					rs4450508	125.1 kb upstream
					rs477181	142.5 kb upstream
					rs502933	142 kb upstream
KCTD15	19	Obesity	GWAS	[65,66]	rs11084753	17 kb downstream
					rs29941	4.4 kb downstream
CTNNBL1	20	Obesity	GWAS	[69]	rs6013029	Intronic
					rs16986921	Intronic
					rs6020712	Intronic
					rs6020846	Intronic
					rs6020395	Intronic
					rs16986890	Intronic
					rs6096781	26.7 kb downstream
					rs6020339	Intronic

### Genotyping

Genotyping was performed with the MassARRAY and iPlex systems of the Sequenom genotyping platform (Sequenom, San Diego, CA), which uses the MALDI-TOF primer extension assay [28,29] according to manufacturers' recommendations.

Assays were optimized in 24 samples consisting of 20 reference Centre d'Etude du Polymorphisme Humain (CEPH) samples and 4 blanks.

All sample plates contained cases, controls, blanks, CEPH and duplicate samples. Quality control measures included independent double genotyping, blind to sample identity and blind to the other caller, and where available comparison of our CEPH genotypes to those in the HapMap http://www.hapmap.org.

### Statistical analysis

SNPs were tested for deviation from Hardy Weinberg Equilibrium (HWE) in both cases (SGA) and controls (AGA), and for the weighted population using a chi-square goodness-of-fit test.

To determine if there were differences between children born SGA and those born AGA, genotype and allele frequencies for each SNP were analyzed by logistic regression using the major allele as the reference for allele analyses and the major homozygote group as the reference for the genotype analysis. Odds ratios show the increased risk (OR > 1) or decreased risk (OR < 1) for the minor allele, or genotype group, of being SGA in relation to the reference group. Univariable logistic regression was carried out to assess the relationship of each SNP with SGA and those found to be significant at the 10% level were carried through to multivariable analyses. The multivariable model controlled for the previously published model for SGA in this population [14] namely gestational age, gender, socio-economic status, age mother left school, marital status, attendance at antenatal classes, primiparity, maternal smoking during pregnancy, marijuana use during pregnancy, maternal height and weight, maternal age at index pregnancy and maternal hypertension.

Statistical analyses were carried out using R [30] and SAS (V9.1 SAS Institute., Cary, NC, USA).

## Results

We do not have genotypic data for 10 SNPs: rs1387153 (*MTNR1B*), rs6020339 (*CTNNBL1*), rs2388399 (*PFKP*), rs477181 (*MC4R*), rs3820152 (*ADIPOR1*), rs7561317 (*TMEM18*), rs7647305 (*ETV5 - DGKG*), rs2844479 (*NCR3 - AIF1*), rs8050136 (*FTO*) and rs10487818 (*NAMPT*). These SNPs either could not be multiplexed into our sequenom assays, failed or did not pass our quality control measure for inclusion in the analysis. The remaining 54 SNPs produced genotypic data for analysis. Each of these SNPs had a genotyping call rate greater than 90% and the genotyping calls did not differ significantly from HWE criteria.

Nine SNPs were significant at the 10% level in the univariable analysis at either the genotypic or allelic level and each of these SNPs was then taken forward to multivariable analysis (see tables 2 and 3 for results).

**Table 2:** Univariable and multivariable associations of obesity and diabetes SNPs with small for gestational age at term (allelic and genotypic) data

								Univariable analysis				Multivariable analysis*			
Gene	SNP	alleles	Minor allele	Risk allele associated with SGA in ABC samples	Risk allele associated with obesity/diabetes	Minor allele frequency	Minor allele homozygote frequency	genotypic p-value	allelic p-value	Odds ratio	95% CI	Genotypic p-value	allelic p-value	Odds ratio	95% CI
															
FTO	rs9939609	AT	A	A	A (obesity)	33.8	11.4	**0.03**	0.42	1.11	0.86-1.43	0.40	0.63	1.07	0.80-1.44
JAZF1	rs864745	AG	G	G	A (diabetes)	48.3	26.7	0.09	0.64	1.06	0.83-1.36	0.26	0.98	1	0.75-1.32
KCNJ11	rs5219	CT	T	T	T (diabetes)	33.7	12.3	**0.02**	**0.03**	**1.32**	**1.03-1.70**	0.061	**0.03**	**1.36**	**1.02-1.82**
LPL	rs3200218	GA	G	A	A (obesity)	26.3	5.5	0.20	0.06	0.76	0.56-1.02	0.21	0.09	0.74	0.53-1.04
PFKP	rs6602024	GA	A	G	A (obesity)	12.0	0	0.08	0.13	0.73	0.49-1.10	**0.03**	0.06	0.64	0.40-1.02
PTER	rs10508503	CT	T	C	C (obesity)	9.8	0.5	**0.04**	0.06	0.64	0.41-1.02	0.07	**0.04**	**0.58**	**0.34-0.98**
SEC16B	rs10913469	TC	C	T	C (obesity)	18.0	1.6	**0.05**	0.82	0.96	0.70-1.33	**0.01**	0.22	0.79	0.54-1.15
BDNF	rs6265	AG	A	A	G (obesity)	16.2	2.2	0.11	0.10	1.33	0.95-1.85	0.10	0.07	1.43	0.98-2.09
BDNF	rs925946	GT	T	G	T (obesity)	30.7	11.1	**0.01**	0.08	0.79	0.61-1.02	**0.043**	0.06	0.75	0.56-1.01

*Multivariable model controls for gestational age, gender, socio-economic status, age mother left school, marital status, attendance at antenatal classes, primiparity, maternal smoking during pregnancy, marijuana use during pregnancy, maternal height and weight, maternal age at index pregnancy and maternal hypertension

**Table 3:** Univariable and multivariable associations of obesity and diabetes SNPs with small for gestational age at term (genotype data)

SNP	genotype	n	Univariable OR	Multivariable* OR
rs9939609	A	61	0.91 (0.50 - 1.64)	0.96 (0.47 - 1.94)
FTO	AT	265	**1.54 (1.07 - 2.22)**	1.30 (0.85 - 1.99)
	T	218	Ref	Ref
				
rs864745	A	145	Ref	Ref
JAZF1	AG	346	**1.57 (1.03 - 2.39)**	1.43 (0.88 - 2.32)
	G	136	1.18 (0.73 - 1.91)	1.04 (0.59 - 1.83)
				
rs5219	C	223	Ref	Ref
KCNJ11	CT	252	**1.64 (1.13 - 2.37)**	1.50 (0.98 - 2.31)
	T	76	1.59 (0.94 - 2.69)	**1.90 (1.04 - 3.50)**
				
rs6265	A	17	**2.84 (1.03 - 7.87)**	**3.29 (1.08 - 9.99)**
BDNF	AG	142	1.17 (0.79 - 1.74)	1.18 (0.74 - 1.90)
	G	347	Ref	Ref
				
rs925946	G	232	Ref	Ref
BDNF	T	66	0.84 (0.48 -1.46)	0.71 (0.38 - 1.34)
	GT	249	**0.58 (0.40 -0.83)**	**0.58 (0.38 - 0.89)**
				
rs6602024	A	0	undefined	undefined
PFKP	GA	116	0.68 (0.44 - 1.04)	**0.57 (0.35 - 0.95)**
	G	435	Ref	Ref
				
rs10508503	C	447	Ref	Ref
PTER	CT	82	**0.55 (0.33 - 0.91)**	**0.51 (0.28 - 0.91)**
	T	3	2.49 (0.22 - 27.7)	1.19 (0.09 -16.58)
				
rs10913469	C	13	2.94 (0.89 - 9.73)	2.54 (0.65 - 9.97)
SEC16B	TC	367	0.74 (0.50 - 1.09)	**0.56 (0.35 - 0.89)**
	T	158	Ref	Ref

* Multivariable model controls for gestational age, gender, socio-economic status, age mother left school, marital status, attendance at antenatal classes, primiparity, maternal smoking during pregnancy, marijuana use during pregnancy, maternal height and weight, maternal age at index pregnancy and maternal hypertension.

Six SNPs demonstrated statistical significance at p < 0.05 in either the univariable or multivariable analysis: rs9939609 which is an intronic SNP in *FTO*, rs5219 which is a missense (Lys-Glu) SNP in *KCNJ11*, rs925946 which is located 9,240 bp from *BDNF*, rs6602024 which is an intronic SNP in *PFKP*, rs10508503 which is located 179,016 bp from *PTER *and rs10913469 which is an intronic SNP in *SEC16B*.

As we have two SNPs in *BDNF *and these two SNPs are in LD (D' = 1, r^2 ^= 0.116) and exist within the same haplotype block (confirmed using haploview 4.2) we conducted a haplotype analysis to determine if the haplotype would give stronger results than the individual SNPs (data not shown). The haplotype analysis of rs6265 and rs925946 found an overall effect (Global Stat = 5.92, p = 0.05). In univariable analysis compared to the GG haplotype the AG haplotype did not show a statistically increased risk (OR = 1.22, 95%CI = 0.86-1.68), whilst those with the GT haplotype had a borderline decreased risk (OR = 0.78, 95%CI = 0.59-1.04). In multivariable analyses these odds ratios moved towards unity and were not statistically significant suggesting that the haplotype analysis does not add anything further to the analysis of each SNP individually.

We have examined anthropometric characteristics of the children at 11 years of age (table 4) and found that the children born SGA remain significantly lighter, shorter and still have lower BMI than the AGA children. Hence not all of the SGA children have shown catch up growth.

**Table 4:** Anthropometric characteristics of children at 11 years of age.

	SGA	AGA	T (p-value)
**Weight (kg)**	40.6 (10.5)	43.9 (10.3)	3.85 (< 0.0001)

**Weight SDS^†^**	0.26 (1.14)	0.71 (1.04)	4.80 (< 0.0001)

**Height (cm)**	147.2 (7.6)	150.9 (7.4)	6.06 (< 0.0001)

**Height SDS^†^**	0.16 (1.07)	0.70 (1.04)	5.92 (< 0.0001)

**BMI**	18.6 (3.7)	19.1 (3.2)	1.98 (0.048)

**BMI SDS^††^**	0.22 (1.22)	0.50 (1.10)	2.91 (0.004)

^**†**^Weight and Height SDS derived from the UK standards of Freeman et al [77]

**^††^**BMI SDS derived from the UK standards of Cole et al [78]

## Discussion

We have found associations (with p values less than 0.05) for SGA with the diabetes related SNP in KCNJ11 and the obesity related SNPs in FTO, PFKP, PTER, SEC16B and BDNF. After controlling for potential confounders the association with the FTO SNP did not remain significant, whilst the other 5 SNPs were positively associated with SGA in the multivariable model.

The T allele of KCNJ11 SNP rs5219 is associated with type 2 diabetes in adults and has been shown to be associated with reduced insulin secretion (see table 1 for references) so our study result finding that this risk allele is associated with being SGA is compatible with the fetal insulin hypothesis where genetically mediated reduced insulin secretion beginning in-utero results in reduced birthweight, and later increases the risk of developing T2 D. Two previous studies evaluated the diabetes related *KCNJ11 *variant with birthweight and found no association [31,32]. It is possible that this variant interacts with other factors, either genetic or environmental, that exist within the ABC cohort but are not present in the other two studies.

Our study found that the high risk allele for obesity in the PTER SNP (C allele at rs10508503) was associated with being SGA. This finding would fit with the fetal insulin hypothesis only if this allele had a direct effect on increasing insulin resistance prior to manifesting as increased BMI later in life, and thus manifests as low birth weight and later leads to obesity. Alternatively, the association of this obesity gene in SGA babies may be due to some survival advantage of being a "thin-fat" baby in terms of inappropriate fat mass for body size [33], not discerned by the simple measure of birth weight. It may be that most of these SGA babies grow into genetically predisposed obese children and adults; hence we are observing the association with post-natal obesity in our SGA cohort.

Conversely, our study found an association of SGA with the low risk alleles for obesity in the BDNF and SEC16B genes suggesting that these alleles may confer a propensity to small size beginning in-utero, since the same SNP in BDNF has been associated with thinness in women [34]. It would be interesting to examine whether this sub-group of SGA babies go on to have improved metabolic outcomes later in life by having a lower risk of obesity.

Since we began this study associations between common variants in type 2 diabetes susceptibility genes have been tested in several large birthweight cohorts. Freathy *et al*, 2009 [35] looked at five type 2 diabetes susceptibility genes and found that the *CDKAL1 *and *HHEX-IDE *loci were associated with reduced birth weight. They did not detect an association with *CDKN2A/B*, *IGF2BP2*, and *SLC30A8*. All 5 of these loci were included in our study and we did not detect an association for any of them with SGA. Zhao *et al*, 2009 [36] also observed an association between lower birth weight and the *CDKAL1 *locus. However, no association was found with 19 other diabetes genes examined, including *KCNJ11 *for which we found an association with SGA. Pulizzi *et al*, 2009 [37] investigated 9 diabetes genes, all of which are included in our study but were not found to be associated with SGA. Of the tested variants, the risk variant in *HHEX *showed a trend towards a low birthweight and the risk variant in the *CDKN2A/2B *locus was associated with high birthweight. The three studies described above investigated birthweight. Only *TCF7L2 *has been studied for association with SGA [38,39]. The gene was not associated with SGA in these two cohorts or our own. However, an association has been described between *TCF7L2 *and birthweight, although the effect was strongest with maternal genotype and after adjustment for maternal genotype fetal *TCF7L2 *genotype was not associated with birth weight [40].

We examined the publically available British 1958 birth cohort database http://www.b58cgene.sgul.ac.uk/ for our significant genes. SNPs in *BDNF *and *FTO *were associated with birthweight but *KCNJ11, PTER, PFKP *and *SEC16B *did not show any associations.

The failure to replicate the associations reported by Freathy *et al*, Pulizzi *et al *and Zhao *et al *and our reporting of significant results for different genes may be due to the different phenotype used (birthweight vs. SGA) and/or due to different study populations with different environmental and genetic influences.

To summarise, we have identified five SNPs/genes which are associated with SGA. While noting that replication in independent samples is essential, our data provides evidence that genetic variation in type 2 diabetes and obesity susceptibility genes such as *KCNJ11, BDNF, PFKP, PTER *and *SEC16B *have a possible role in SGA as well as their established roles in obesity and/or diabetes.

We recognise that the association observed with these SNPs are unlikely to survive any adjustment for multiple testing and could thus be false positives. But it is possible that we may be seeing small genetic effects here and as our sample size is small compared to the majority of genetic association studies today we have low power to detect these associations with a high level of statistical significance. Calculations of statistical power using PS 2.1.31 [41] show that for the ABC study we have 31.61% power to detect an odds ratio of 1.2, 56.84% power to detect an odds ratio of 1.3 and 78.01% power to detect an odds ratio of 1.4 for a SNP with a minor allele frequency of 0.48 (such as rs864745 in JAZF11). For SNPs with a lower allele frequency the power would be less. For example for a SNP with a minor allele frequency of 0.12 (such as rs6602024 in PFKP) we have 17.36% power to detect an odds ratio of 1.2, 31.42% power to detect an odds ratio of 1.3 and 48.05% power to detect an odds ratio of 1.4.

It is also possible that these genes may have more subtle effects and could affect a related phenotype, rather than be directly associated with SGA. Although beyond the scope of this paper it would be interesting to look at these SNPs/genes in relation such phenotypes e.g., catch up growth. Alternatively, the associations could reflect underlying LD with other markers in these genes. Further analysis in these genes with which we demonstrate an association with SGA is therefore required. Also, further investigation of the 36 genes for which we found no association should not be ruled out. The lack of association of these genes with SGA in our sample could be explained by a lack of power and we cannot rule out that we were unable to detect smaller effects of these variants. It is also possible that these obesity and/or diabetes genes may lead to small decreases in birthweight but do not result in the more severe SGA phenotype. Alternatively, it may be possible that any direct effects of susceptibility genes resulting in an individual being born SGA (by reduced insulin secretion) may be offset by an opposing effect from the maternal genotype (through the effects of the same variants on maternal glucose levels) [42]. Unfortunately, maternal DNA samples are not presently available from the ABC cohort and so we are unable to test this.

During revision of this manuscript Freathy and colleagues reported a meta-analysis of genome-wide association studies and followed up the top hits in 13 replication studies [43]. They identified two loci, in *ADCY5 *and near *CCNL1*, that are associated with birth weight and explain 0.3% and 0.1% of the variance in birth weight, respectively. Both loci were also associated with smallness for gestational age. SNPs in *ADCY5 *have recently been implicated in regulation of glucose levels and susceptibility to type 2 diabetes [44], providing further evidence that the association between lower birth weight and/or SGA and subsequent type 2 diabetes does indeed have a genetic component.

## Conclusion

In conclusion, this study supports the concept that genetic factors associated with obesity and/or risk of type 2 diabetes are more prevalent in those born SGA compared to those born AGA. We have previously determined that maternal diet during pregnancy [15] and other environmental factors [14] are associated with differences in birthweight in the ABC study and now we have demonstrated a significant genetic contribution, suggesting that it is most likely that there is an interaction between the genetic determinants of birthweight, childhood growth and risk of adult metabolic diseases with both the intra- and extra-uterine environments.

## Abbreviations

ABC: Auckland Birthweight Collaborative; SGA: Small for gestational age; SNPs: Single nucleotide polymorphisms; ABC: Auckland Birthweight Collaborative; AGA: Appropriate for gestational age; CEPH: Centre d'Etude du Polymorphisme Humain; HWE: Hardy Weinberg Equilibrium; MALDI-TOF: Matrix Assisted Laser Desorption/Ionization - Time of Flight.

## Competing interests

The authors declare that they have no competing interests.

## Authors' contributions

ARM made substantial contributions to this study. She identified genes to investigate, designed and carried out the genotyping experiments and was primary author of the manuscript. JMDT was responsible for the analysis and interpretation of data and contributed to the writing of the manuscript. He also helped in establishing the ABC cohort and its data collection. RM participated in the writing of the manuscript. PNB gave advice on study design and participated in the writing of the manuscript. WJL was responsible for DNA extraction and also assisted with the genotyping experiments. LRF participated in study design and coordination, and in editing of the manuscript. EAM conceived the study, and participated in its design and coordination. He also established the ABC cohort and its data collection. All authors read and approved the final manuscript.

## Pre-publication history

The pre-publication history for this paper can be accessed here:

http://www.biomedcentral.com/1471-2350/11/125/prepub
